# Letter from Dr. Nash

**Published:** 1875-07

**Authors:** James A. Hart


### EDITORIAL. ETC.

---

The following letter, with the teeth enclosed, was received from Dr. James A. Nash, of Vienna, Georgia, and is the history of a very remarkable case, somewhat similar in its features to the one related by the late Dr. Andrews, of Charlotte, N. C., which appeared in one of the early numbers of the 2nd Series of this JOURNAL. The specimens have been placed in the Museum of the Baltimore College of Dental Surgery, and consist of a cuspid and bicuspid of normal size and form, and quite a number of malformed teeth, some of which are exceedingly small, and all with more or less enamel on the misshapen crowns ; several appear to be clusters of small teeth joined in one mass :

"On Jan. 15th, 1874, I was called in to see a Miss Fannie Murray, aged seventeen, daughter of one Mr. Jas. Murray ; found the right side of the face very much swollen. The cuspid and first bicuspid on the same side were missing, and the remainder of the teeth in a tolerably healthy condition and formed naturally.

My first diagnosis was abscess in the antrum of Highmore, and I prepared to puncture it, but soon discovered an abnormal growth of imperfectly developed teeth with diseased bone—the bone being in a necrosed condition. After dissecting up the gum, I removed the imperfect teeth, fifteen in number, and very small, and a considerable amount of diseased bone. Puncturing the antrum for further disease, there came at least one ounce of yellow fetid watery fluid, with nearly one-fourth the amount of pus. Upon further examination, I found a tooth of the natural size embedded in the antrum that I removed—it proved to be the first permanent bicuspid—I did no more at that time.

March 1st. I called to see the lady and found by the swollen parts that the disease was not entirely cured. I removed another root, some more bone, and by thoroughly examining with a probe and using a trocar, I found another tooth embedded in the bone at an angle of about forty-five degrees with the nose, and situated just below the infra-orbital foramen. This I removed, and it proved to be a cuspid of natural size and well formed.

Saw the lady again on the 18th of March, and found her doing well—the gums healed by first intention, &c.

I have since seen the lady, and she is permanently cured and not disfigured."

James A. Hart.

